# Centimeter-wide worm-like fossils from the lowest Cambrian of South China

**DOI:** 10.1038/s41598-017-15089-y

**Published:** 2017-11-06

**Authors:** Xingliang Zhang, Wei Liu, Yukio Isozaki, Tomohiko Sato

**Affiliations:** 1 State Key Laboratory of the Continental Dynamics, Shaanxi Key Laboratory of Early Life and Environment, Department of Geology, Northwest University, Xi’an, 710069 PR China; 2 Department of Earth Science and Astronomy, University of Tokyo, Tokyo, 153-8902 Japan

## Abstract

The trace fossil record implies that large worm-like animals were in place along with the skeletonizing organisms during the initial stage of the Cambrian explosion. Body fossils of large worms, however, have so far not been found. Here, we describe a large, soft-bodied, worm-like organism, *Vittatusivermis annularius* gen. et sp. nov. from the lowest Cambrian of South China, which is constrained to the Fortunian Age (541–529 Ma) of the Cambrian Period. The elongate body of *Vittatusivermis* was large enough to have supported organ systems and a fluid skeleton that facilitated peristaltic locomotion, thus allowing for more complex patterns of movement than those of flatworms. Its occurrence on the same bedding surface as trace fossils suggests that *Vittatusivermis* might have produced epichnial trails and shallow burrows on and within sediments. Therefore, *Vittatusivermis* is likely to have been one of the long expected producers of trace fossils in the earliest Cambrian.

## Introduction

The Cambrian explosion was an evolutionary event of great magnitude, as evidenced by the abrupt appearances of diverse animal lineages in the fossil record during the early Cambrian (~541–509 Ma)^1–4^. Tubes, shells, and sclerites of small shelly faunas are characteristic fossils of the Terreneuvian Epoch (~541–521 Ma)^5,6^. Exceptionally preserved soft-bodied faunas in subsequent Cambrian Epoch 2 (~521–509 Ma) reveal a more complete faunal composition of the Cambrian explosion^7,8^. Trace fossils, however, offer an independent line of evidence supporting the explosive nature of the Cambrian event^9^. The presence of large burrows and trails (1 cm or more in diameter) implies that soft-bodied, worm-like animals with a hydrostatic skeleton evolved immediately before the earliest Cambrian. However, no confirmed body fossils that could have produced such trace fossils have as yet been found before or during the Terreneuvian Epoch, largely because macroscopic soft-bodied faunas are absent from this period.

Here, we report a large, soft-bodied, worm-like organism, *Vittatusivermis annularius* gen. et sp. nov., from lowest Cambrian phosphoritic rocks of South China, which are constrained to a Fortunian (541–529 Ma) age (see below). The vermiform body of *Vittatusivermis* is annulated, approximately one centimeter or more in width, and more than 26 centimeters in maximum preserved length. In gross morphology, the fossil resembles worm-like animals of the Bilateria. Anatomic details, however, are not preserved. The co-occurrence of trace fossils on the same bedding surface in the immediate vicinity of body fossils as well as specimens interpreted to have been preserved in burrowing position hint that *Vittatusivermis annularius* might have been an active trace producer. If correct, our finding would provide a potential trace maker of contemporary traces such as *Psammichnites*.

## Results

### Geological setting

**Figure 1:**
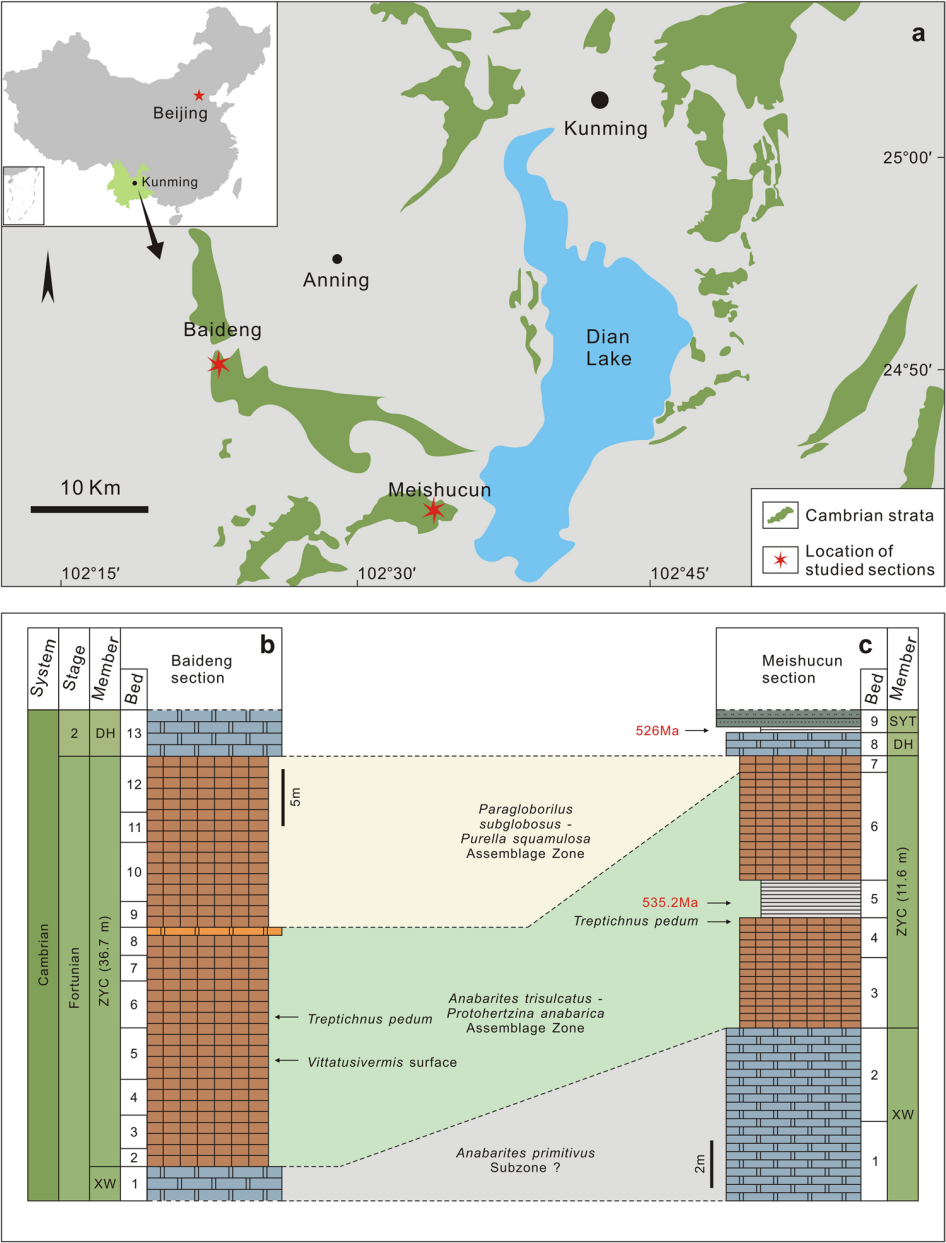
Locality, stratigraphy and age of the *Vittatusivermis* bed. (**a**) Distribution of Cambrian outcrops. (**b**) and (**c**) Litho- and biostratigraphy of studied sections. DH, Dahai Member, SYT, Shiyantou Member, XW, Xiaowaitoushan Member, and ZYC, Zhongyicun Member. The map was created using CorelDRAWX4 (Copyright 2008 X. Zhang and Corel Corporation. All rights reserved).

All specimens occur in the Zhongyicun Member of the Yuhucun Formation, on the same surface of phosphoritic Bed 5^10^ of the Baideng section, located in the Tianning Phosphorite Mine, Anning County, Yunnan Province, China (Fig. 1). The Baideng section was measured and studied in detail by Luo *et al*.^10^. The Zhongyicun Member of the Yuhucun Formation is 36.7 m thick, and is referred to as the “Baideng-Beds 2–12”^10^. The fossil bearing bed is about 8 m above the base of the Zhongyicun Member, corresponding to Baideng-Bed 5 of Luo *et al*.^10^, which is extremely fossiliferous with abundant small shelly fossils. The index fossil *Anabarites trisulcatus* which was known to range from Beds 2 to 8 in the Baideng section^10^ is abundant on the fossil surface (Supplementary Fig. 1b,c). This indicates that the *Vittatusivermis*-bearing surface is well within the *Anabarites trisulcatus*– *Protohertzina anabarica* Assemblage Zone^11,12^. Slightly above this surface, the basal Cambrian index fossil *Treptichnus pedum* (Supplementary Fig. 1a) was found within the Baideng-Bed 6^10^. The lowest occurrence of *T*. *pedum* in South China was previously reported from the uppermost part of the lower phosphorite unit at the Meishucun section^13,14^ (Fig. 1).

The Meishucun section in Jinning County, ca. 25 km southeast of the Beideng section (Fig. 1), was once considered as a candidate section for the basal Cambrian GSSP and thus has been intensively studied. The Zhongyicun Member here is 11.6 m thick and comprises Meishucun-Beds 3–7^10^. Two biozones were recognized as the *Anabarites trisulcatus*– *Protohertzina anabarica* Assemblage Zone (Meishucun-Beds 3–6) and the *Paragloborilus subglobosus*–*Purella squamulosa* Assemblage Zone (Meishucun-Bed 7)^11,12^. A prominent, so-called “white clay” (Meishucun-Bed 5), a dolomitic and fine-grained clastic unit interbedded with a bentonitic clay, divides the Zhongyicun Member into lower (Meishucun-Beds 3–4) and upper (Meishucun-Beds 6–7) phosphorite units. This bentonite is widely thought to be an altered volcanic tuff which has been dated and revised several times by different working groups, with the estimated ages varying widely between 525–539 Ma^15–20^. More recently the depositional age of the bentonitic clay was suggested to be approximately 535.2 ± 1.7 Ma^20^. Additionally, an ash from Meishucun-Bed 9, nearly at the base of the Shiyantou Member, was dated at ca. 526.5 ± 1.1 Ma^18^. The lowest occurrence of *Treptichnus pedum* occurs in the uppermost part of the lower phosphorite unit in the Meishucun section^14^.

Bed to bed correlation between the two sections is not possible and the bentonitic clay has not been recognized in the Baideng section. Hence, it is difficult to determine whether the *Vittatusivermis* surface pre-dates or post-dates the 535 Ma bentonitic clay. However, as described above, the *Vittatusivermis* surface can be confidently placed within the middle part of the *Anabarites trisulcatus*– *Protohertzina anabarica* Assemblage Zone, probably below the lowest occurrence of *Treptichnus pedum* in South China. Therefore, its age is estimated to be early Fortunian, close to 535 Ma.

### Preservation

**Figure 2:**
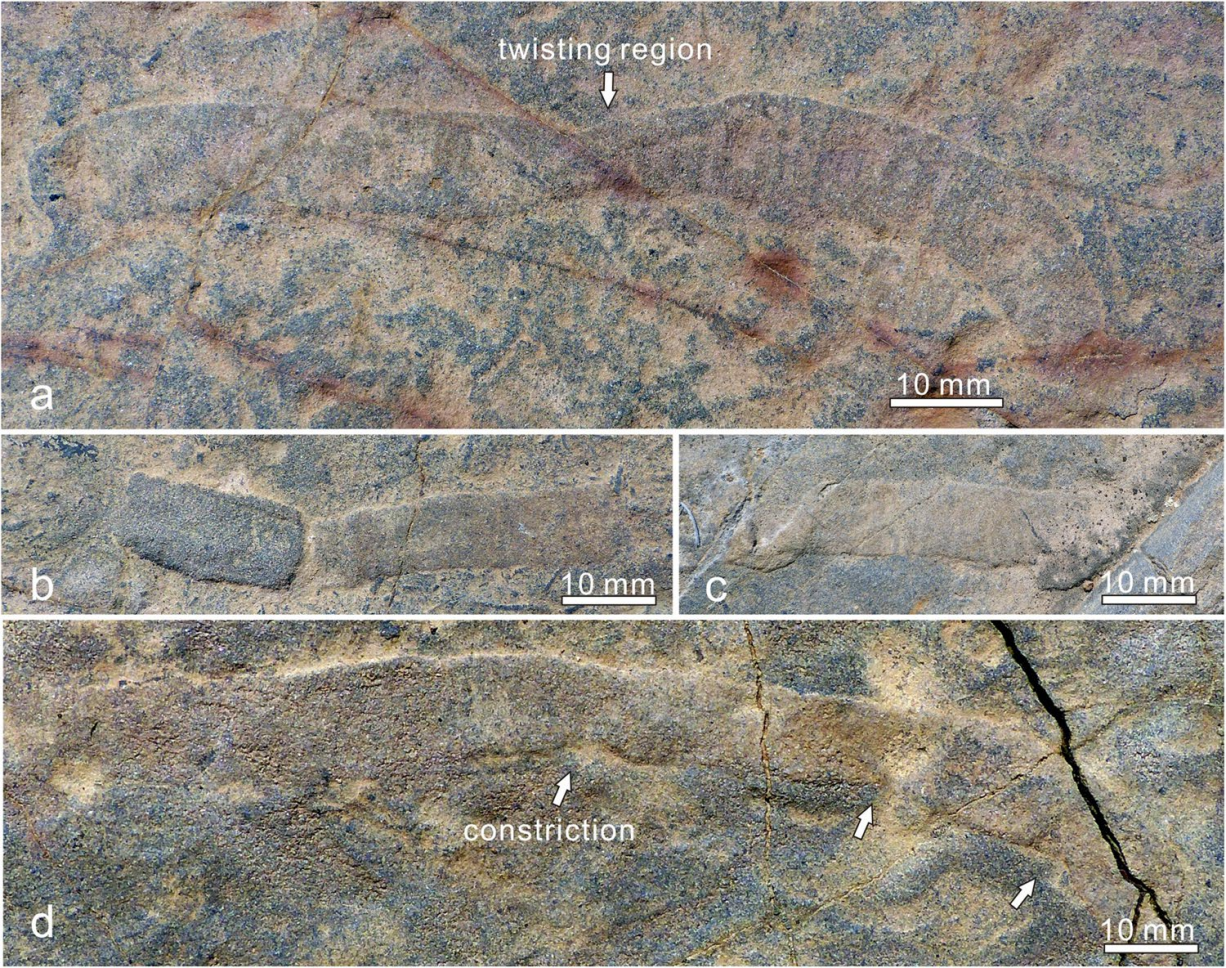
*Vittatusivermis annularius* gen. et sp. nov. from the lowest Cambrian of the Baideng section, Anning, Yunnan, South China. (**a**) L1070477, holotype, twisted 180 degrees at midlength, resulting in a considerable constriction in body width, cross annulations visible in the right portion. (**b**) L1030133, bent slightly, body width constricted on the right side. (**c**) L1030117, showing a twist at the left end. (**d**) L1030160, bent slightly, with regional constrictions (white arrows).

**Figure 3:**
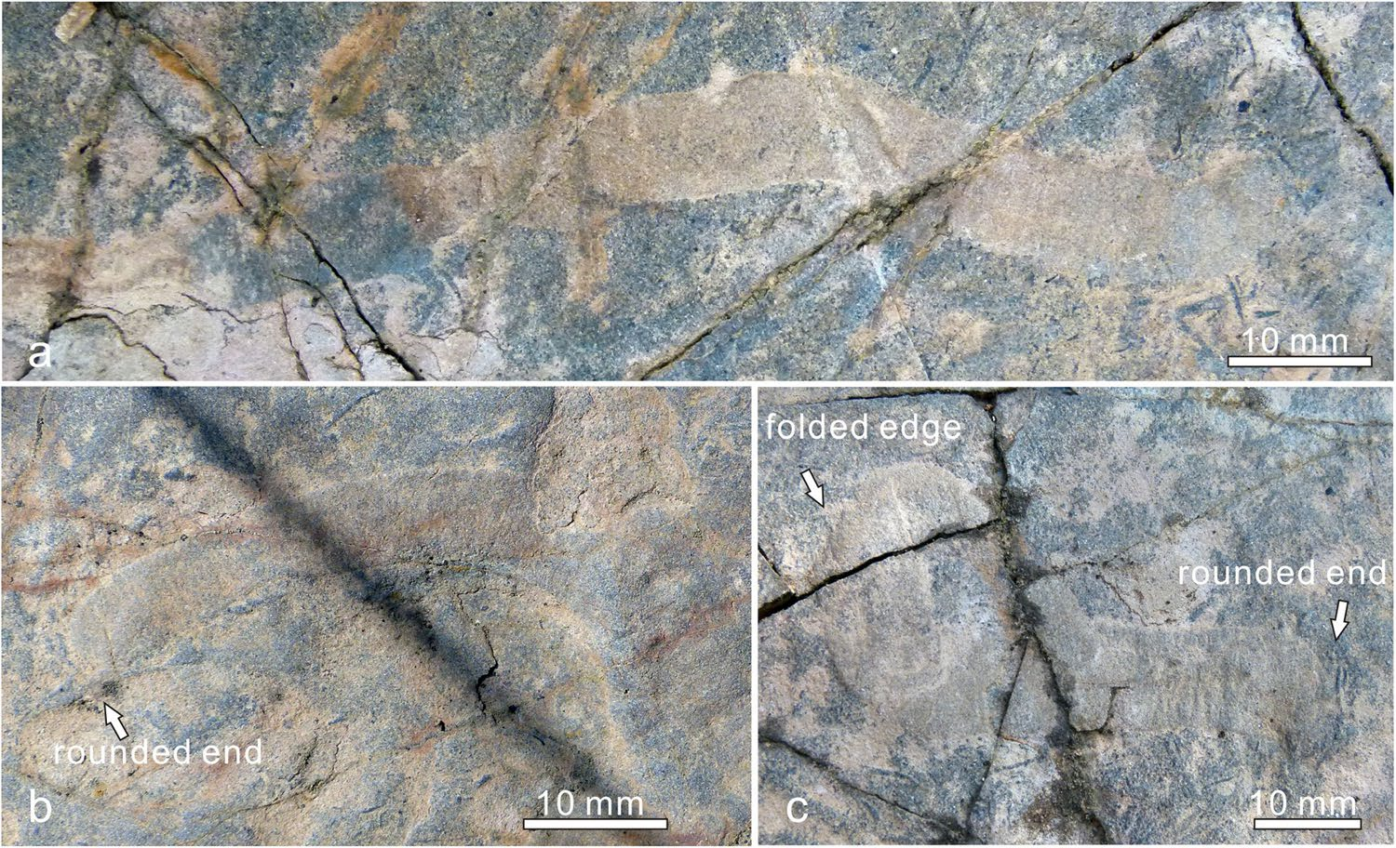
*Vittatusivermis annularius* gen. et sp. nov. from the lowest Cambrian of the Baideng section, Anning, Yunnan, South China, showing curvature in different degrees. (**a**) L1070410, gently curved into an arc, showing a rounded end at the right. (**b**) L1030186, curved into a semicircle with a rounded end at the left. (**c**) L1070388, tightly curved, showing a folded edge at the upper left and a rounded end at the lower right.

**Figure 4:**
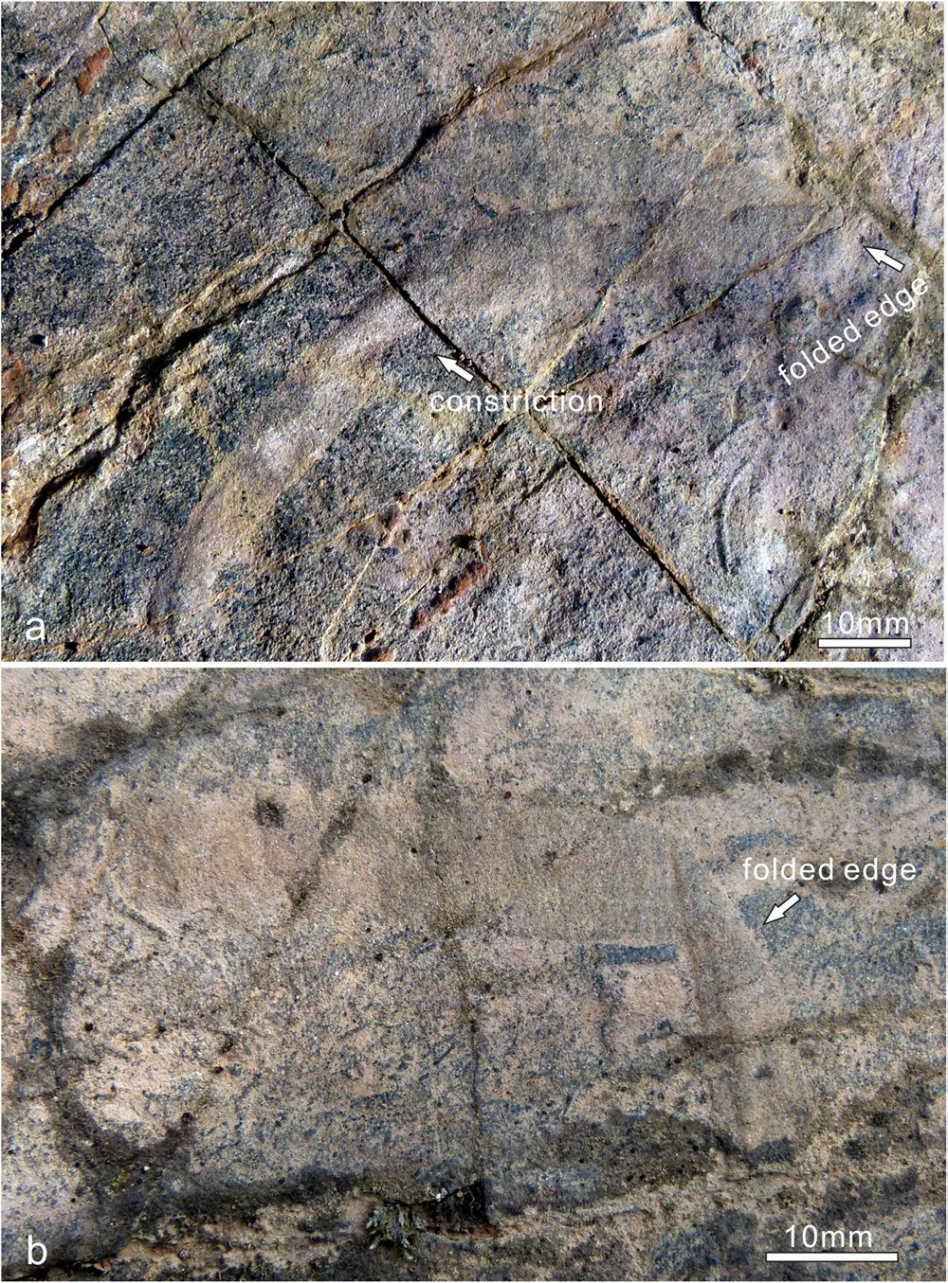
*Vittatusivermis annularius* gen. et sp. nov. from the lowest Cambrian of the Baideng section, Anning, Yunnan, South China, showing folding structures and differentiation of both sides of the flattened body. (**a**) L1070410, folded lengthwise, resulting in a folded edge proximately perpendicular to the body length, the overlapping body part curved and showing a constriction in body width probably originated from a fold along the body length. (**b**) L1070389, folded obliquely, the folded edge intersecting the body length in a obtuse angle.

**Figure 5:**
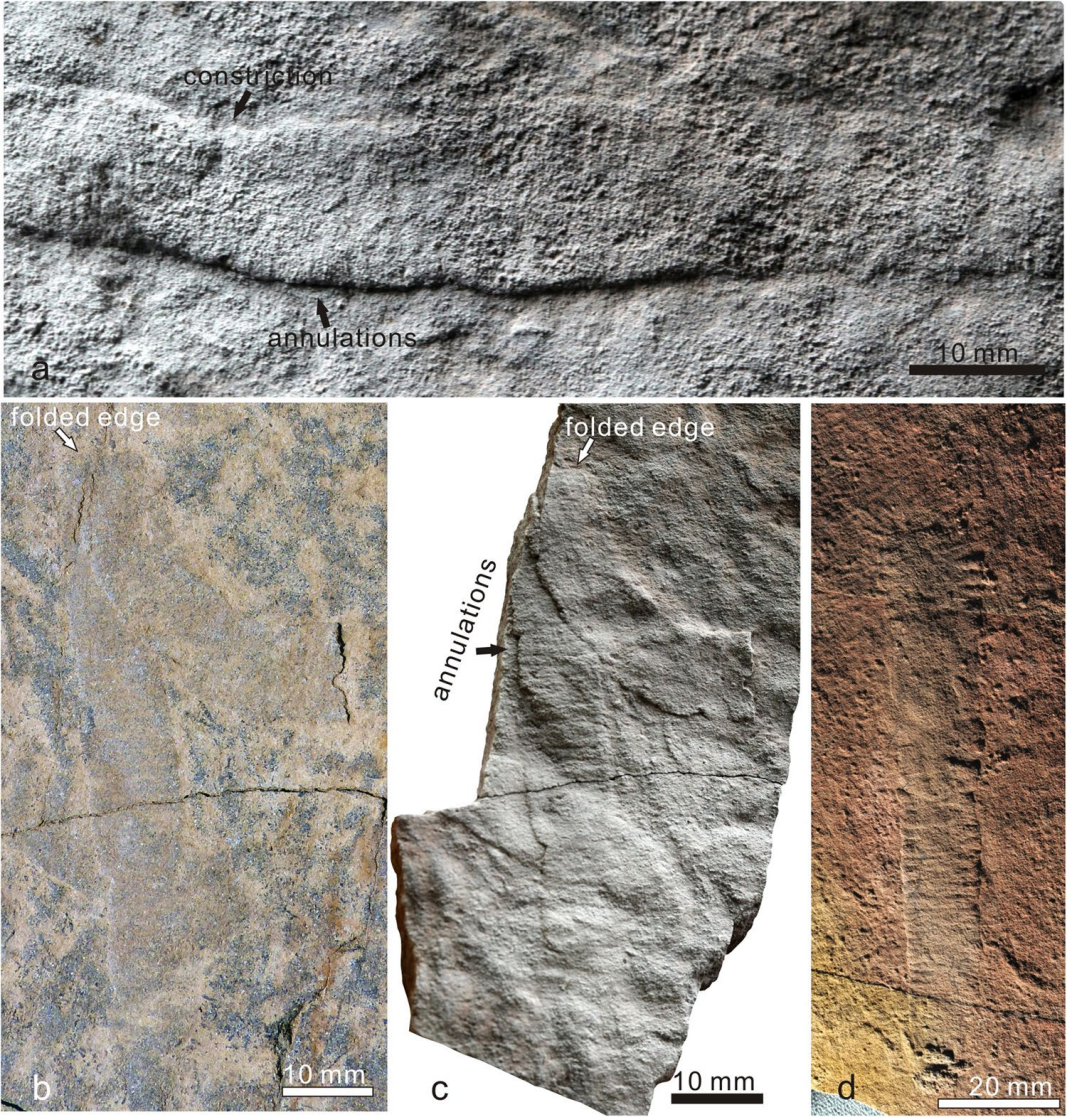
(**a–c**) *Vittatusivermis annularius* from the lowest Cambrian of the Baideng section, Anning, Yunnan, South China, with transverse annulations specifically emphasized. (**a**) L1030160, coated with MgO film and illuminated in a low angle light, showing cross annulations. (**b**) LELE1585, folded lengthwise, showing a folded edge and both sides of the flattened body undifferentiated. (**c**) the same specimen of *b*, coated with MgO film and illuminated in a low angle light, showing cross annulations. (**d**) *Wutubus annularis* from the late Ediacaran of South China, NIGP159084, image courtesy of Zhe Chen.

All specimens of *Vittatusivermis* are preserved as compressions flattened parallel to the bedding surface (Figs 2, 3, 4 and 5, Supplementary Fig. 3). They are randomly distributed on the surface although individuals may occur in clusters (Supplementary Figs 2, 3). Plastic deformation such as bending, overfolding, twisting, and body constriction are widespread among specimens (Figs 2–5), which indicate *Vittatusivermis* is a soft-bodied organism. Rapid burial is assumed for the preservation of soft tissues, although this assumption remains to be confirmed by detailed palaeoenvironmental and sedimentological analyses.

Thin section examination shows the *Vittatusivermis* bed is comprised of phosphatic grainstones (Supplementary Fig. 4a) with subordinate terrigenous clasts. Terrigenous clasts are predominantly fine quartz sands, making up ca. 15% of total grains. The remaining 85% are phosphatic, and include phosphatized peloids, bioclasts, and fragments of microbial mats, which vary from fine to coarse sand-sized. The grains were cemented by silica minerals, mostly chert and chalcedony. Surprisingly, abundant microbial remains, including spherical and filamentous forms, which are most conspicuous under epifluorescent illumination (Supplementary Fig. 4c,d), have been found within cements. The exquisite preservation of interstitially colonized microbial communities suggests that the cementation took place during early diagenesis.

Collectively, the above analyses suggest rapid burial and rapid lithification of the *Vittatusivermis* bed, both of which contributed to the preservation of *Vittatusivermis*. The rapid burial protected the organism from deterioration by biotic and/or abiotic processes, while the rapid lithification ascribed to early diagenetic cementation ultimately captured the form of the organism on the bedding surface.

**Systematic Palaeontology**.

? Bilateria

Phylum uncertain

Genus *Vittatusivermis* gen. nov.

*Type and only known species*. *Vittatusivermis annularius* gen. et sp. nov.

*Etymology*. Derived from Latin, *vittatus* (ribbon-shaped) and *vermis* (worm), referring to the ribbon-shaped and worm-like body. Gender masculine.

*Diagnosis*. The vermiform body is flexible, elongated, and dorsoventrally or laterally flattened (ribbon-shaped). Both ends are rounded. The width, one centimeter or more, is consistent throughout the entire preserved length (reaching 26 centimeters in maximum). The body surface is ornamented with tightly arranged transverse annuli.

*Vittatusivermis annularius* sp. nov.

*Etymology*. Derived from Latin, *annularius*, in reference to the transverse annuli on the surface of the ribbon. Gender masculine.

*Holotype*. Holotype is referred to the specimen L1070477 (Fig. 2a).

*Other material*. An additional 65 specimens are preserved on the same surface at the Tianning Phosphorite Mine Quarry, which were measured and photographed.

*Locality and horizon*. Tianning Phisphorite Mine Quarry at Baideng village, Anning County, Yunnan. Lower part of the Zhongyicun Member, Yuhucun Formation, Cambrian Terreneuvian Series and Fortunian Stage, *Anabarites trisulcatus*– *Protohertzina anabarica* Assemblage Zone.

*Diagnosis*. As for the genus.

*Description*. Specimens have been found at the top surface of a coarse-grained phosphoric grainstone bed. They are preserved as elongated compressions with low relief (Figs 2–5, Supplementary Fig. 3). Mapping of the fossil surface shows that specimens are randomly distributed and have no specific orientation (Supplementary Fig. 2). Specimens are preserved as flattened ribbons which vary from 8–18 mm in width and reach 26 cm in maximum preserved length (Supplementary Table 1). The width of most specimens is almost always consistent throughout the preserved length, except for a minor proportion of specimens which show regional constrictions originating from plastic deformation (e.g. Figs 2a–d, 3a). The anterior and posterior of the organism are not able to be defined as anatomical features are not preserved. A number of specimens show rounded ends (Fig. 3). One-third of specimens occur as straight compressions, and the remainder of specimens show plastic deformations in various postures (Supplementary Table 1), indicating that they are the remains of a soft-bodied organism rather than trace fossils. Bodies are found twisted, bent, coiled, and folded at different attitudes (Figs 2–5, Supplementary Fig. 3). The holotype is twisted at its midlength, resulting in a constriction in this region (Fig. 2a). Bent specimens show different degrees of curvature, varying from gently curved arcs (Fig. 3a) to semicircles (Figs 3b, 4a and 5b) and to tightly curved coils (Fig. 3c). Specimens occur folded in lengthwise or at an angle perpendicular or oblique to the long axis of the body (Fig. 4). All folded specimens have a sharp and straight folded edge (Figs 3c, 4a,b and 5b,c), from which it can be reasonably inferred that the worm-like body was flattened either dorsoventrally or laterally. Alternatively, the flattened ribbons could likely represent moults of a worm-like organism with a round cross section. Folded specimens also indicate that the sides of the flattened body are not differentiated from one another (Figs 4a,b, 5b,c). Tightly arranged transverse annulations, about five per centimeter, are present in some specimens (e.g. Figs 2a, 5a–c).

**Figure 6:**
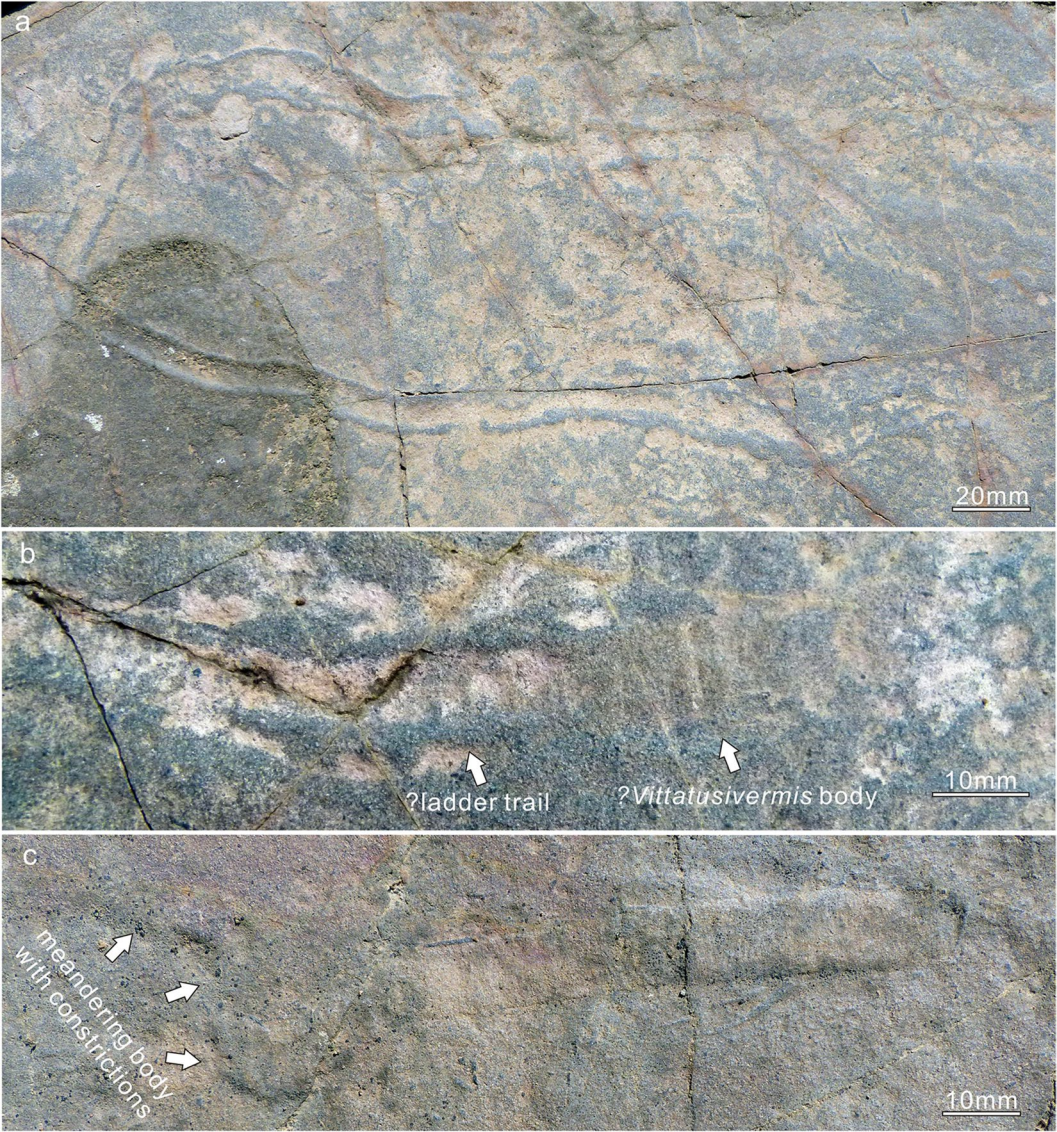
Trace fossils occurring with *Vittatusivermis annularius* gen. et sp. nov. on the same bedding surface. (**a**) L1030181, a bilobate epichnial trail assigned to the *Plagiogmus*-preservation of *Psammichnites*, appearing as a “ribbon trail” on the upper bedding surface. (**b**) L1070465, the left part showing a bilobate trail with regularly spaced cross ridges, comparable to the basal “ladder trail” of the *Plagiogmus*-preservation, while the right half representing partially preserved *Vittatusivermis* body with regularly arranged cross annulations. (**c**) L1030204, the left part meandering and constricted, suggesting a burrowing behavior.

*Remarks*. As discussed below, trace fossils are abundant on the *Vittatusivermis* surface. However, they are readily distinguished from body fossils by their style of preservation. Traces are preserved in concave epirelief with marginal ridges (Fig. 6a), whereas specimens assigned to *Vittatusivermis* occur as flattened ribbons with low, positive relief (Fig. 5a–c). Furthermore, plastic deformation is a very common phenomenon, in particular, the folding structure, which is unlikely for trace fossils.

Although it cannot be placed in any known genus, in overall form *Vittatusivermis* is similar to a number of late Ediacaran and early Cambrian soft-bodied, tubular fossils with transverse annulations, e.g. *Gaojiashania*, *Onuphionella*, *Sabellidites*, *Shaanxilithes*, *and Wutubus* ^21–27^. *Vittatusivermis* resembles the basal part of *Wutubus* ^21^ (Fig. 5d) from the late Ediacaran of South China, however, the latter is conotubular in shape, circular in cross section, and exhibits regional differentiation (apex and tube). Additionally, specimens of *Wutubus* illustrated by Chen *et al*. (2014, Figs 5–7)^21^ are preserved as straight tubes without plastic deformation. General resemblances also exist between *Vittatusivermis* and the tube *Gaojiashania* (late Ediacaran, South China)^22^, but the rigid ring-flexible bucket tube architecture is not recognized in *Vittatusivermis*. It is worth noting that two tubular specimens with cross annulations described in Smith *et al*. (2016, Fig. 2A and B)^27^ from the latest Ediacaran of the Deep Spring Formation, Nevada, USA, are very similar to *Vittatusivermis* in shape and dimension. However, these two specimens have no terminal ends preserved and were assigned to *Gaojiashania* ^27^. *Onuphionella*, *Sabellidites*, and *Shaanxilithes* are morphologically very similar. They are elongate tubes with a constant diameter along the entire length of the fossil, and have closely spaced cross annulations or corrugations^23–26^. However, these three fossils are typically smaller in diameter and lack rounded terminations as ween in *Vittatusivermis* (Fig. 3). The phylogenetic position of these elongate tubes is problematic because diagnostic features are not preserved and many unrelated organisms may develop encasing tubes independently. An exception is the tubular fossils *Sabellidites*, as the microstructure of its organically persevered tube wall was interpreted as analogous to that of annelid siboglinids^26^.

## Discussion

### Affinities

The relatively simple morphology of these fossils makes it difficult to assign *Vittatusivermis* to a specific clade. This is a fairly common phenomenon in the body fossil record of the earliest animals, a few representatives of known groups are present, but most of the record is of uncertain affinity^28^. The macroscopic size and morphological differentiation between individuals of *Vittatusivermis* clearly indicate that it is a multicellular organism. Although basal metazoans like sponges and cnidarian-grade animals may also include tubular forms, the worm-like body with annulations and association with bilaterian-produced trace fossils may suggest that *Vittatusivermis* was a soft-bodied bilaterian. However, *Vittatusivermis* lacks either anterior-posterior (or ventral-dorsal) differentiation or preserved internal anatomical features, and the bilaterian affinity of this fossil cannot be confirmed independently by its potential association with trace fossils on the same surface (see discussion below). Therefore, we tentatively place *Vittatusivermis* within the Bilateria. Fluid-filled worm-like forms are widely distributed across bilaterian clades, e.g. enteropneusts of the Deuterostomia, priapulans of the Ecdysozoa, and annelids of the Lophotrochzoa.

Among modern marine bilaterians, nemerteans and flatworms are dorsoventrally flattened worms, but the former tend to be more robust and elongate than the later. In addition, the epidermis of some nemertean species is annulated^29^. These characters are consistent with a nemertean interpretation. However, the proboscis apparatus, which is unique to nemerteans and represents a novel synapomorphy distinguishing the Nemertea from all other invertebrate taxa^29,30^, is not preserved in *Vittatusivermis* specimens. *Vittatusivermis* is also broadly similar to some groups of annelids, notably the leeches which are markedly flattened and have a large number of segments with superficial annulations, but more diagnostic features, e.g. the anterior and posterior suckers of leeches, are not recognized in *Vittatusivermis*.

**Figure 7:**
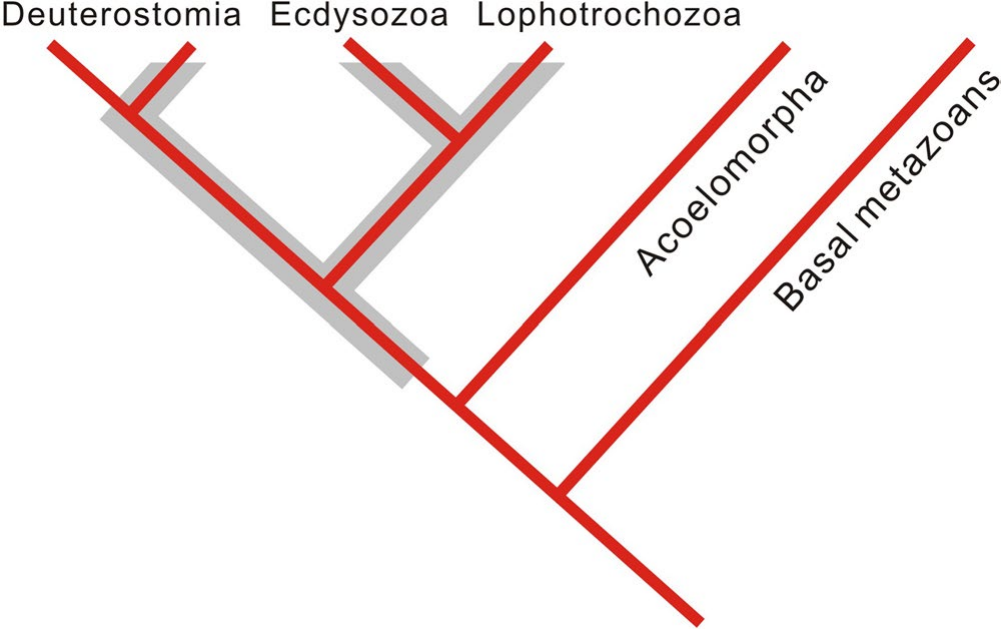
A metazoan phylogeny showing possible placements of *Vittatusivermis annularius* (grey envelope).

The plastic deformations preserved in *Vittatusivermis* specimens (Figs 2–5) indicate it is a soft-bodied organism. However, its epidermis might have been enhanced by a cuticle, which gives it a well-defined border as was seen in each specimen. Therefore, it is also likely that the specimens of *Vittatusivermis* comprise the remains of molted cuticles. The Ecdysozoa has received its name from the fact that all its representatives molt their cuticles. However, cuticles evolved independently several times among bilaterial lineages, and molting alone is not a sufficient character to support the monophyly of the Ecdysozoa because some annelids (e.g. the leech *Hirudo medicinalis*) are known to molt their cuticlar structures as well^31^. Similarly, the presence of cross annulations in *Vittatusivermis* has little phylogenetic implication because annulations are very widespread among metazoans and could evolve convergently. Consequently, *Vittatusivermis* can most parsimoniously be considered a worm-like organism lying somewhere on the tree of bilateral invertebrates above the Acoelomorpha (Fig. 7).

### Trace fossil associations

A number of trace fossils were previously reported from the Zhongyicun Member, revealing a wide variety of behavioural patterns, including branching burrow systems (e.g. *Treptichnus* and *Sellaulichnus*), systematic guided meanders (e.g. *Psammichites*), and arthropod traces (e.g. *Rusophycus*)^32–34^.

On the *Vittatusivermis*-bearing surface, a large bilobate meandering trail is present (Fig. 6a), approximately 50 cm from the closest body fossil. The trace is approximately one centimeter wide and is preserved as a concave epirelief structure, showing that two sharp, parallel marginal ridges are separated by a wide, shallow, and smooth trough (Fig. 6a). The trail is reminiscent of *Taphrhelminthopsis cirularis* from the stratigraphically higher Shiyantou Member^32^. This form was subsequently reinterpreted as a preservational variant of *Psammichnites* ^33^. Another bilobate specimen with regularly spaced cross ridges (much thicker and less closely spaced than body annulations) is immediately connected with a piece of *Vittatusivermis* body with regularly arranged cross annulations (Fig. 6b). Both are comparable to components of the *Plagiomus*-style preservation of *Psammichnites*, the former corresponding to the upper bedding surface “ribbon trail” and the later to the basal “ladder trail”^35,36^.

With regard to the taxonomic affiliation of the *Psammichnites* trace maker, it has been speculated that a slug-like animal related to halkieriids without dorsal armors could produce such trace by passing through sediments^37,38^. However, the co-occurrence of traces and *Vittatusivermis* body fossils on the same bedding surface suggests that *Vittatusivermis* is also likely to be a *Psammichnites* maker.

### Life mode

**Figure 8:**
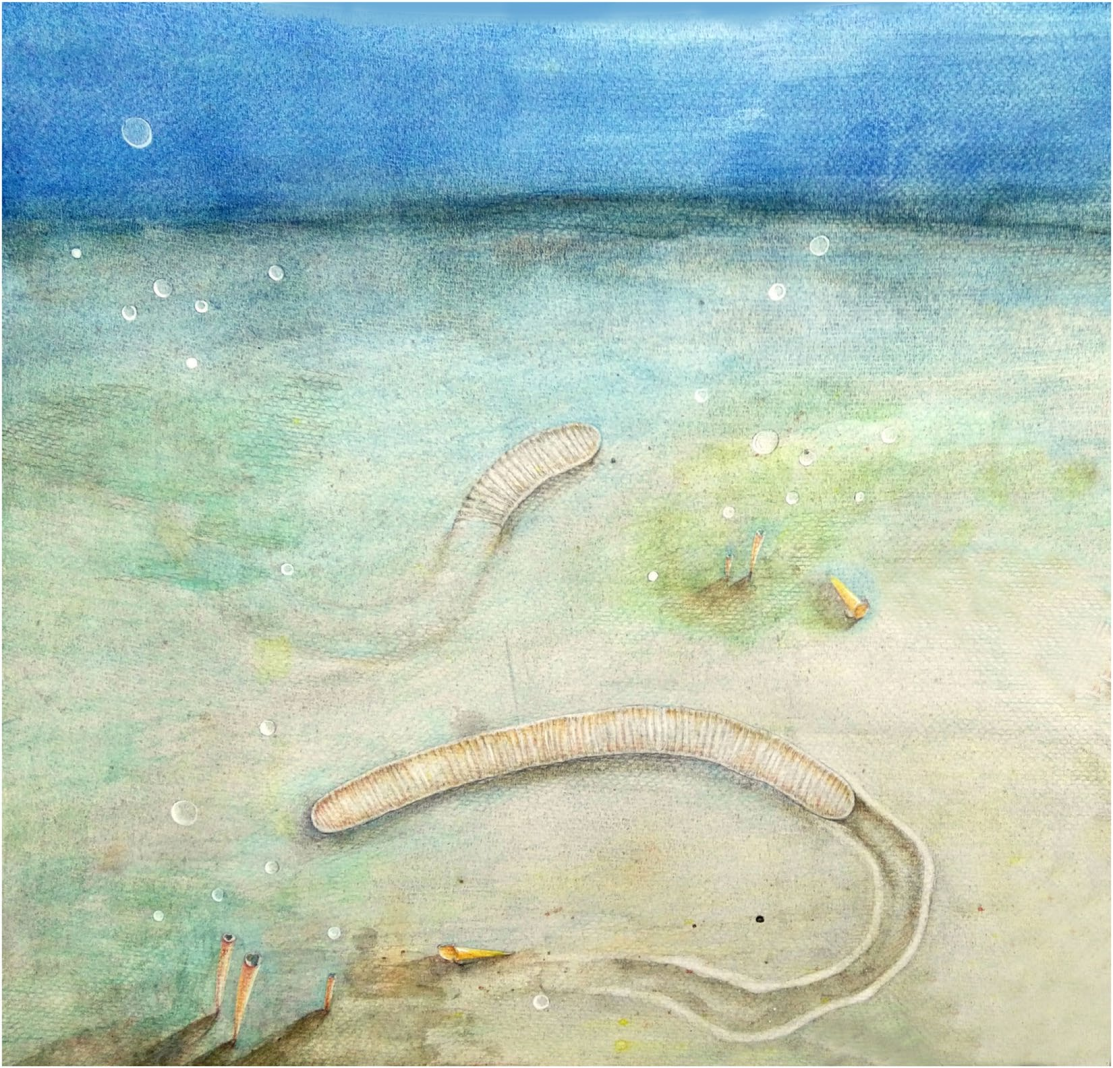
Ecosystem reconstruction of the *Vittatusivermis* bed, showing *Vittatusivermis annularius* as an active trace maker.

*Vittatusivermis* is large enough to have organ systems and thus is inferred to be capable of making traces comparable in dimension and complexity to the trace fossils with which it is found. The worm-like body with annulations can be analogized to vermiform animals across many bilateral lineages, which employ coelomic spaces as a hydrostatic skeleton for support and locomotion (Fig. 8). Specimens preserved in a variety of postures (Figs 2–5; Supplementary Figs 2, 3; Supplementary Table 1) suggest that the organism was able to twist and coil, while the trace fossil association indicates that it may have engaged in epibenthic and burrowing locomotory patterns.

In the absence of locomotory appendages and other body outgrowths, it is most likely that *Vittatusivermis* used muscular waves to crawl over the soft substratum, and, in doing so produced a trail. When burrowing, it may have employed peristatic waves of body wall muscles to propel it through soft substrates. Unlike some burrowing polychaetes, which anchor the body by extending the chaete laterally from the buried segments^39^, *Vittatusivermis* may have burrowed through sediments with the surface of the expanded portions of the body serving as anchor points, while the burrow wall as an antagonistic force resisting the hydraulic pressure. The peristatic waves would thus have moved constricted body regions forward while the anchored parts provided leverage. The presence of constricted body regions in some specimens (e.g. Figs 2b,d, 6c) is also suggestive of such burrowing behaviors. Intriguingly, the left portion of the specimen in Fig. 6c is curving and constricted in width, while the right portion is straight and expanded, which gives an indication that the animal was likely killed in the act of burrowing.

Therefore, it is reasonable to assume that *Vittatusivermis* was a bottom dweller and an active trace maker. The associated trace fossils (Fig. 6a), interpreted here as *Plagiogmus*-style preservation of *Psammichites*, were likely produced by *Vittatusivermis*. It is unclear whether *Vittatusivermis* was able to swim from one place to another or leave the substratum as a short-term mechanism to escape benthic predators.

## Conclusion

The metazoan fossil record of the earliest Cambrian, during the Fortunian Age of the Terreneuvian Epoch, is marked by the appearance of distinctly larger, more complex trace fossils than those of the terminal Ediacaran and by the appearance of the relatively abundant small shelly fossil fauna. It is evidently during this interval that the disparate bodyplans of many bilaterian lineages evolved, although most were represented by stem groups^28^. Early Cambrian traces include significantly larger, more diverse, and more complex forms than their Ediacaran predecessors^9,39^. The presence of horizontal, infaunal traces suggests the presence of vermiform animals with fluid skeletons to facilitate peristaltic locomotion. However, body fossils of macroscopic, soft-bodied animals have not previously been found among faunas of the Fortunian Age. Microscopic, soft-bodied vermiforms *Eopriapulites* and *Eokinorhynchus*, representing stem group priapulan and kinorhynch forms, respectively, were reported from the contemporaneous small shelly fauna of South China^40,41^, but they are too minute to have produced the larger trails or burrows observed throughout the Fortunian rock record. The newly discovered *Vittatusivermis* is a centimeter-wide, soft-bodied organism. Its macroscopic size and worm-like appearance are consistent with expectations of the contemporaneous trace fossil record. This, together with its close spatial association with traces, allows a tentative interpretation of *Vittatusivermis* as a soft-bodied bilaterian, placed somewhere above the Acoelomorpha within the phylogenic tree of the Metazoa, and thus is a potential producer of traces found in the earliest Cambrian. Therefore, the discovery of *Vittatusivermis* has significant implications for faunal evolution during the initial stage of the Cambrian explosion. It supports the suggestion from the trace fossil record that macroscopic, soft-bodied faunas evolved along with the advent of shelly faunas at the beginning of the Cambrian Period. Such soft-bodied macrofauna may be preserved under proper taphonomic conditions and additional examples from the Fortunian remain to be found in the future.

## Methods

A total of 66 specimens of *Vittatusivermis annularius* were found associated with two specimens of the trace fossil *Psammichnites* (identified as *Plagiomus*-preservation and *Taphrhelminthopsis*-preservation, respectively) on the same bedding surface (named the *Vittatusivermis* surface) which was exposed approximately 32 square meters. The bedding surface was mapped in 50 cm × 50 cm grids to show the patterns of distribution of specimens and their relationship to trace fossils (Supplementary Fig. 2). Most of the specimens were left *in situ* in the Tianning Phosphorite Mine Quarry located at Baiden Village, Anning County, Yunnan Province (Fig. 1), and protected by the Tianning Phosphorite Mine Company. Three specimens were sampled for further analyses in laboratory and are reposited at Department of Geology, Northwest University, Xian, China. All specimens were measured (Supplementary Table 1) and photographed under sun light in the field. The sampled specimens were coated with a MgO film and photographed under low angle illumination to enhance annulations on the surface (e.g. Fig. 5). Thin sections were made and examined under petrographic microscopy for petrographic, palaeontological, and taphonomic analyses (Supplementary Figs 1b,c, and 4).

## Electronic supplementary material

Supplementary Information
