# Supplementary material for: Centimeter-wide worm-like fossils from the lowest Cambrian of South China
Source: Sci Rep. 2017 Nov 6;7:14504. doi: 10.1038/s41598-017-15089-y (PMC5674079; doi:10.1038/s41598-017-15089-y)
Supplement: Supplementary file 1 — Supplementary Information [file 41598_2017_15089_MOESM1_ESM.pdf]

Centimeter-wide worm-like fossils from the lowest Cambrian of South China

Xingliang Zhang[1]*, Wei Liu[1], Yukio Isozaki[2], Tomohiko Sato[2]

[1]State Key Laboratory of the Continental Dynamics, Shaanxi Key Laboratory of Early Life and Environment, Department of Geology, Northwest University, Xi'an 710069, PR China.

[2]Department of Earth Science and Astronomy, University of Tokyo, Tokyo 153-8902, Japan.

*Correspondence and requests for materials should be addressed to X.Z. (email: xzhang69@nwu.edu.cn).

Supplementary Figures

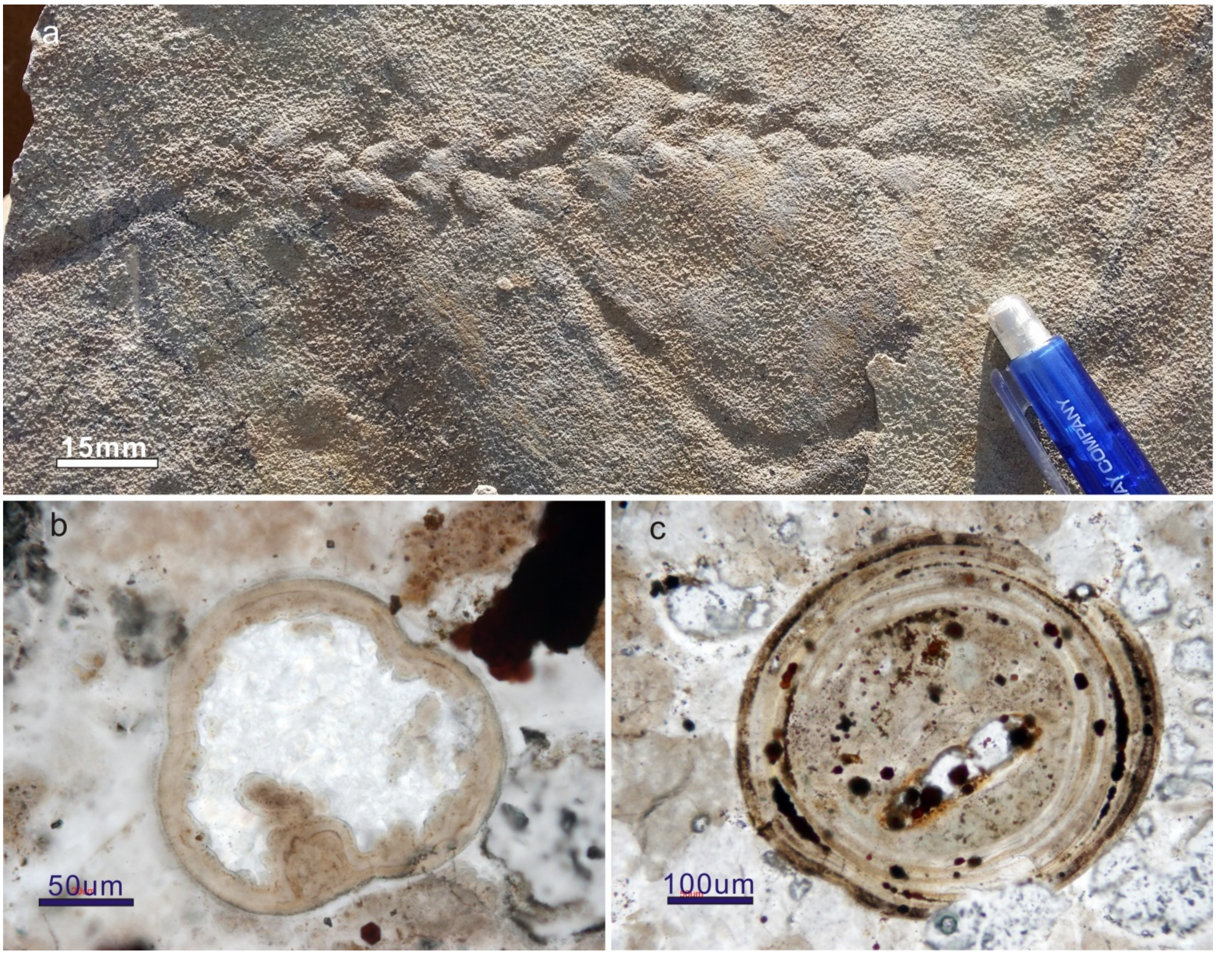

Supplementary Figure 1 | Fossils with biostratigraphical importance from the Baideng section. a, *Treptichnus pedum* from the base of Baideng-Bed 6, slightly above the worm bed. **b**, Baideng-02-08, cross section of *Anabarites trisulcatus* from the worm bed. **c**, Baideng-01-02, cross section of *Cambrotubulus decurvatus* from the worm bed, showing a tube-in-tube architecture, which is known from the Cambrian Terreneuvian of South China.

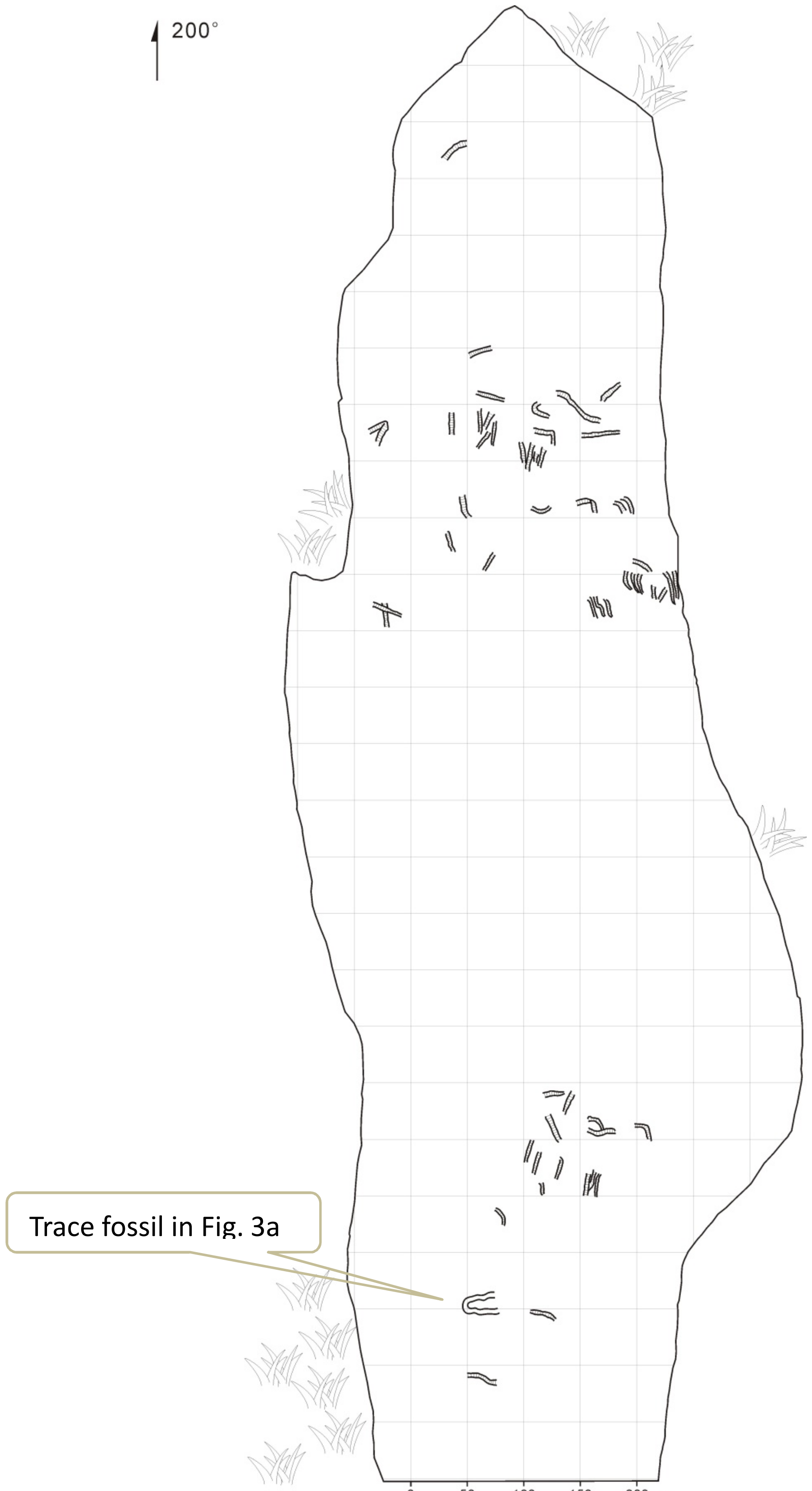

Supplementary Figure 2|Map of the worm surface showing the distribution pattern of specimens. Each grid 50 cm × 50 cm in size and specimens not scaled.

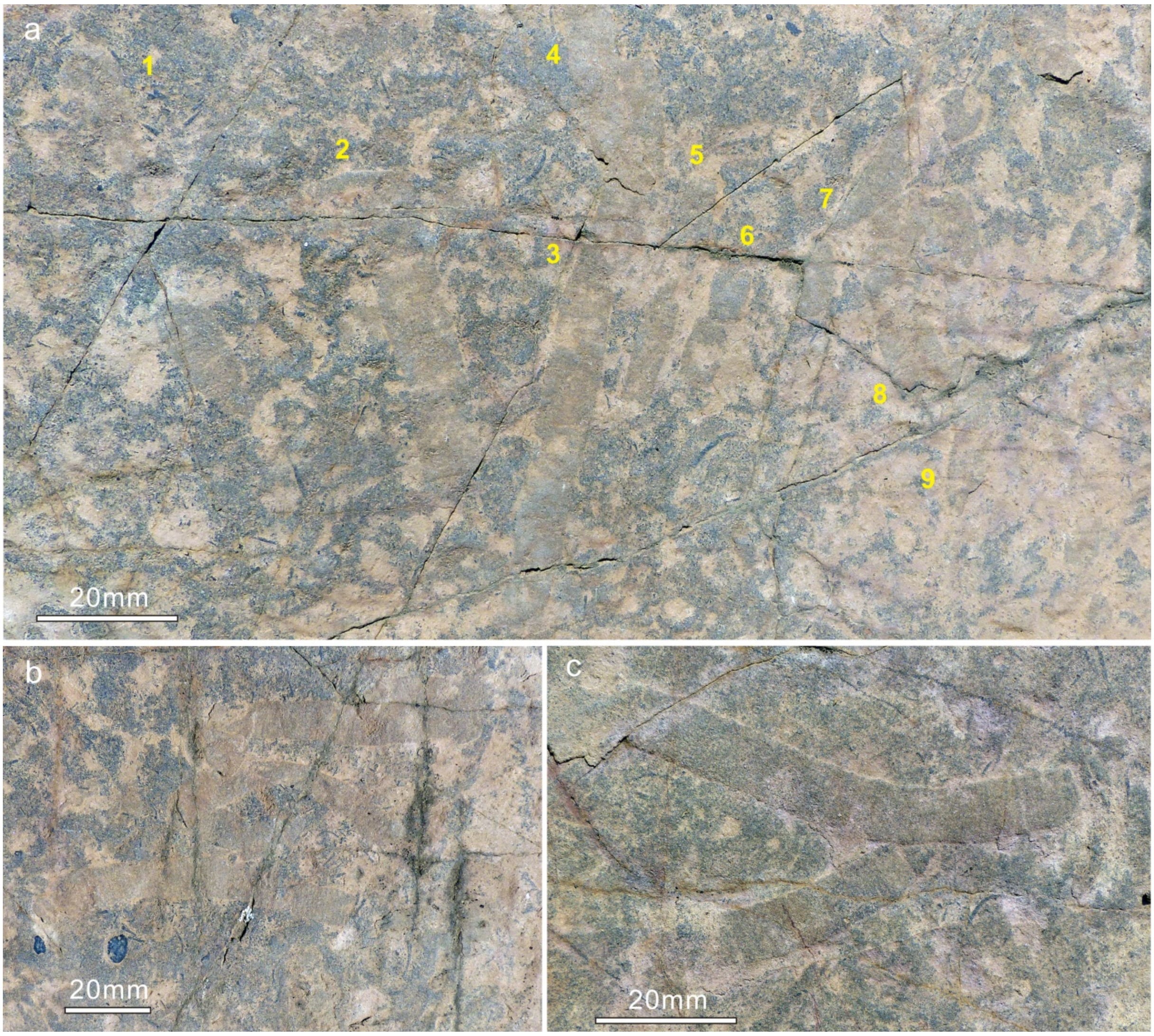

Supplementary Figure 3│*Vittatusivermis annularius* from the worm surface, occurring as clusters. **a**, L1030158, nine specimens indicated in numbers. **b**, L1070404. **c**, L1030198.

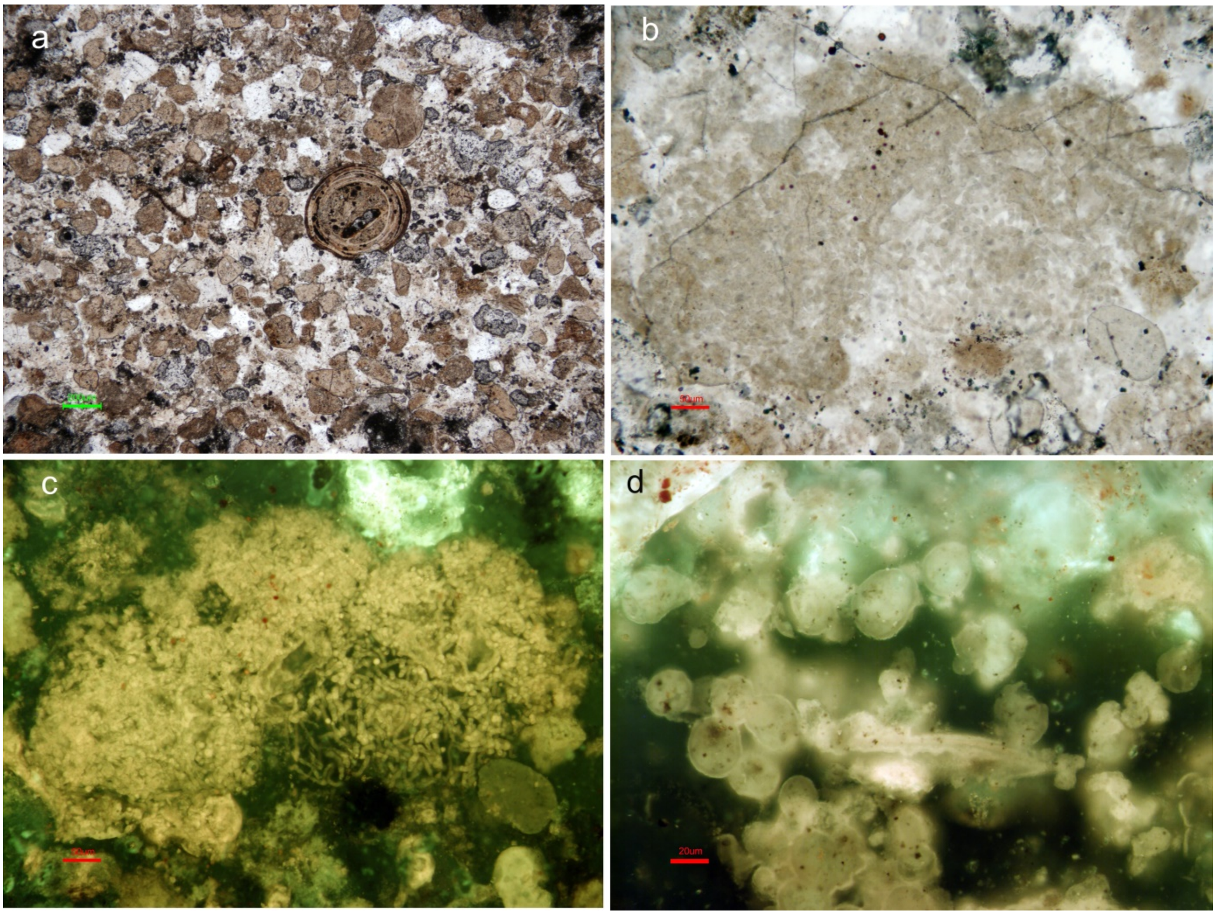

Supplementary Figure 4|**Microphotographs of thin sections of the worm bed**. Microbial remains, bioclasts, and phosphatized peloids are strongly autofluorescent in contrast with dark siliceous cements. Scale bars, 200 µm in **a**, 50 µm in **b** and **c**, 20 µm in **d**. **a**, Baideng-01-01, phosphatized grains and terrigenous clasts are cemented by silica minerals. **b**, Baideng-02-02, interstitially preserved microbial remains. **c**, epifluorescent image of **b**, showing interweaved microbial filaments. **d**, Baideng-06-03, interstitially colonized microbial cocci, epifluorescent illumination.

Supplementary Table 1.

Measurements and preservation postures of specimens.

specimens	length (cm)	maximum width (cm)	minimum width (cm)	preserved posture
LELE1585	10.07	1.53	1.40	Folded
L1030133	7.20	1.41	1.02	Bent, Constriction
L10301581	5.88	1.45	1.23	Straight
L10301582	12.91	1.58	1.33	Folded
L10301583	13.63	1.52	0.92	Folded
L10301584	3.27	1.20	1.11	Bent
L10301585	6.26	1.22	0.77	Straight
L10301586	3.42	0.98	0.92	Straight
L10301587	10.99	1.07	0.78	Folded
L10301588	5.46	1.44	1.10	Bent
L10301589	7.81	1.26	0.85	Bent
L1030160	13.80	1.27	0.88	Bent, Constriction
L1030186	7.36	1.10	0.94	Bent, Constriction
L10301981	7.86	0.88	0.80	Bent
L10301982	7.41	0.92	0.60	Straight, Constriction
L10301983	5.41	1.04	0.80	Bent
L1030204	14.28	1.20	0.95	Bent
L1070379	3.80	1.08	1.08	Straight
L1070382	4.83	1.40	1.19	Straight, Constriction
L1070386	6.55	1.51	1.26	Bent
L1070388	10.67	1.61	0.99	Bent
L1070391	7.59	1.17	1.00	Folded
L1030165	11.20	1.67	1.40	Bent
L1070396	7.29	1.30	0.89	Straight, Constriction
L10704041	8.16	1.23	1.00	Folded
L10704042	10.36	1.15	0.87	Folded , Twisted
L10704043	9.17	1.29	0.99	Bent
L1070410	22.75	1.24	0.68	Folded , Twisted
L1070412	4.35	1.06	0.53	Bent
L10704141	2.73	0.95	0.67	Bent
L10704142	3.86	0.82	0.55	Bent
L1070416	6.57	1.28	0.90	Bent, Constriction
L1070418	6.83	1.32	1.03	Bent
L1070419	6.13	1.53	1.12	Straight
L1070421	8.45	1.04	0.89	Straight, Twisted
L1070424	4.95	1.03	0.98	Straight
L10704261	5.92	1.31	1.07	Straight
L10704262	8.02	1.46	1.18	Bent
L10704263	10.41	1.75	1.61	Straight
L10704264	10.74	1.56	0.86	Straight, Constriction
L10704265	19.08	1.71	1.32	Folded
L10704281	7.00	1.15	1.15	Bent
L10704282	6.20	1.30	1.30	Straight
L10704301	9.38	1.13	1.13	Straight

L10704302	26.78	1.36	0.91	Bent
L10704321	7.33	1.19	1.19	Straight
L10704322	9.04	1.26	1.10	Straight
L10704323	18.44	1.33	1.11	Straight
L10704331	10.69	1.59	1.31	Bent, Constriction
L10704332	11.03	1.60	1.17	Bent
L1070436	4.24	1.05	0.92	Straight, Constriction
L1070438	15.45	1.40	1.19	Folded
L1070440	11.00	1.30	0.50	Bent
L10704431	11.19	1.25	0.80	Bent
L10704432	14.94	1.50	1.19	Bent
L1070445	17.12	1.33	0.44	Bent, Twisted
L1070447	13.68	1.47	0.89	Bent
L1070449	7.74	1.24	0.61	Bent, Constriction
L1070450	22.44	1.79	0.96	Folded, Constriction
L10704511	20.56	1.47	1.13	Bent
L10704512	25.36	1.61	1.24	Bent
L1070455	9.70	1.33	0.96	Straight
L1070459	12.53	1.48	1.30	Bent
L1070465	7.86	1.29	0.98	Straight
L1070477	14.70	1.66	0.85	Bent, Twisted
L107bd001	11.32	1.18	0.96	Bent

--- Supplementary Table 1 continued---
